# “*Dentists are never seen*”: perspectives on multiple job holding among dentists in Nairobi, Kenya

**DOI:** 10.3389/frhs.2025.1595302

**Published:** 2025-07-10

**Authors:** Cyril Ogada, Laetitia C. Rispel

**Affiliations:** ^1^ Centre for Health Policy & South African Research Chairs Initiative, School of Public Health, Faculty of Health Sciences, University of the Witwatersrand, Johannesburg, South Africa; ^2^ Department of Dental Sciences, Faculty of Health Sciences, University of Nairobi, Nairobi, Kenya

**Keywords:** multiple job holding, dentist, oral health, health workforce, Kenya

## Abstract

**Introduction:**

Multiple job holding (MJH), the phenomenon of working in more than one paid job simultaneously, affects the achievement of universal health coverage. The dearth of research on MJH among dentists, especially in Africa, forms the backdrop to this study. This study aimed to explore the perspectives of key policy actors on MJH among dentists in Nairobi, Kenya.

**Methods:**

This qualitative study combined semi-structured interviews with key informants and in-depth interviews with dentists who are engaged in MJH. The key informants were selected purposively from the Kenyan government, the regulator, representative organizations of dentists, and oral health researchers and/or experts in human resources for health. The dentists were selected from the government, the private sector, and faith-based organizations, using snowball sampling. The interviews focused on knowledge and/or experiences of MJH, reasons for, and the consequences of MJH. The interviews were analyzed using thematic analysis.

**Results:**

Thirty interviews were conducted, comprising 20 key informants, and 10 dentists. MJH among dentists is seen as a normative practice, facilitated by a profession characterized by high rewards and few or no adverse consequences from absenteeism. Although additional income is the primary motivation for MJH, low job satisfaction, the lack of continuing professional development, perverse incentives, and a dysfunctional and resource-constrained public health sector exacerbate MJH. The lack of regulation compounds the practice, while a strong private health sector provides opportunities for multiple sources of income, affecting the provision of oral health services negatively in the public sector.

**Conclusion:**

MJH among dentists in Nairobi, Kenya is common because of high rewards and few or no adverse consequences from absenteeism. The high reported occurrence of MJH requires a multi-pronged approach that combines individual, system, and structural interventions. Such an approach should also consider the drivers of MJH, and ensure collaboration among policymakers, dentists, and health service managers to develop strategies to mitigate the potential negative consequences of MJH for patients, the health workforce, and oral healthcare delivery in Nairobi.

## Introduction

The health workforce is central to the achievement of Universal Health Coverage (UHC) (1, 2), which enunciates the global goal of equitable access to quality, comprehensive healthcare services according to need and without suffering financial hardship (3). UHC enables improvements in the health of populations. Dentists play a crucial part in addressing the health needs of populations, as oral health is intricately linked to overall well-being (4). In contrast, compromised oral health adversely affects food choices and nutrition, and is associated with many chronic systemic diseases (5).

Multiple job holding (MJH), defined as working in more than one paid job concurrently, is a complex workforce phenomenon with apparent increases in several industrialized countries (6). MJH is influenced by a combination of socioeconomic, cultural, health system and individual factors, as well as the regulatory environment (7). Globally, there are wide variations in the policy responses of governments to MJH, ranging from a complete ban on the practice, to restrictions with different intensities and regulatory instruments (8, 9). MJH is also influenced by the behaviours and/or actions of policy actors, such as trade unions or professional associations (10), who may endeavour to optimize employment benefits for their members by encouraging flexible policies on MJH (11).

In the health system, MJH is common among health care providers, especially those with scarce skills such as medical specialists (12). The benefits of MJH for individual health professionals include additional income, skills and work diversification, and personal fulfilment (13). However, numerous disadvantages have also been reported including exhaustion, absenteeism, and potential conflicts between the demands of an individual worker's primary and secondary jobs (14, 15).

In the health system, MJH could assist with the retention of health professionals in the public health sector (16). However, MJH has implications for the equity, efficiency, and quality of healthcare provision (8, 17). In low-and middle-income countries (LMICs) MJH, together with weak or no regulation, may exacerbate inequities between the public and private health sectors, and between urban and rural areas (18). These inequities could adversely influence the achievement of UHC (19).

Kenya is an East African country with an estimated population of 51 million. The healthcare system is characterized by a devolved public health care system and a private sector. The private sector is comprised of private-for-profit facilities and faith-based and non-governmental organizations (NGOs) (20). Kenya transitioned into a devolved system of governance in 2013, with transfer of health service delivery to 47 semi-autonomous county governments, while the national government retained health policy and regulatory functions (20, 21). Kenya suffers a net deficit of dentists, with one dentist for every 42,000 people in 2015 (22). This is six-fold below the WHO recommended ratio of one dentist for every 7,000 people.

In LMICs, there is emerging evidence on MJH among health professionals, focusing on its regulation, prevalence, predictors and/or the health system consequences (18, 23–26). However, there is a dearth of studies on MJH among dentists, both globally, but especially in LMICs and in Africa. In addition, the majority of studies focus on doctors and nurses (13, 27), with a novel 2023 MJH study on rehabilitation therapists, in addition to nurses and doctors (28).

This paper aims to bridge this knowledge gap by an in-depth exploration of the perspectives of key policy actors on MJH in the dental profession in Kenya, its influencing factors, and potential consequences. In our study, a policy actor refers to “*a social entity, a person or an organization, able to act on or exert influence on a decision*” (29). There are several reasons for an in-depth, exploratory study on MJH among dentists. First, dentists play a crucial role in safeguarding oral health and well-being by providing essential preventive, curative, and rehabilitative services, and MJH could exacerbate the shortages of dentists in Kenya. Second, in contrast to doctors or nurses who work in different health care settings, oral health care is provided primarily in an ambulatory care setting, which provides an important contextual difference on MJH among dentists. Lastly, by incorporating the perspectives of key policy actors on MJH, this study could contribute to the broader discourse on human resources for health (HRH) and the design of health policies that address MJH, while optimizing the contribution of dentists within Kenya's healthcare system. The latter is important, as there is no legislation or written policy on MJH in Kenya.

## Methods

### Study setting

The setting for this study was Nairobi City County, the financial, commercial and administrative capital of Kenya, and one of the 47 counties (decentralized administrative units) in the country (30, 31). Nairobi is the commercial hub of Eastern Africa and has a multi-ethnic population of about 4.3 million people.

The reasons for selecting Nairobi as the study setting is threefold. Firstly, Nairobi has the highest reported concentration of dentists, with the widest variety in work settings and specializations. For example, Nairobi is host to the largest dental school in Kenya with a large number of dental specialists who work as lecturers/researchers, the largest public referral hospital in Kenya with many dental specialists who work as clinicians, and the largest number and size of both private-for-profit as well as private-not-for profit dental clinics and hospitals. Secondly, Nairobi hosts the headquarters of the Ministry of Health in Kenya where dentists involved with policy are situated, thus enabling recruitment of this population of interest. Lastly, a combination of financial (available budget, study cost) and logistical (location of principal investigator, ease of access) factors also influenced the selected of Nairobi as a study setting.

### Study design

This was an exploratory, qualitative study, that sought to explain “how” and “why” a particular phenomenon, or behaviour, in this case multiple job holding, operates as it does among dentists in Nairobi, Kenya. The qualitative research was part of the formative phase of a larger cross-sectional mixed methods doctoral study that focuses on MJH and the professional quality of life of dentists in Nairobi, Kenya. The qualitative research preceded the planning of a large, quantitative survey among dentists.

### Participant selection

We used purposive sampling to select 20 key informants based on their knowledge and/or experience of MJH, HRH, oral health, and/or the health system in Kenya. We also used a combination of purposive and snowball sampling to select 10 dentists who have previously done or are engaging in MJH. The dentists included both generalists and specialists and working in the public sector, private-for-profit sector, and faith-based or non-governmental organizations.

**Figure 1:**
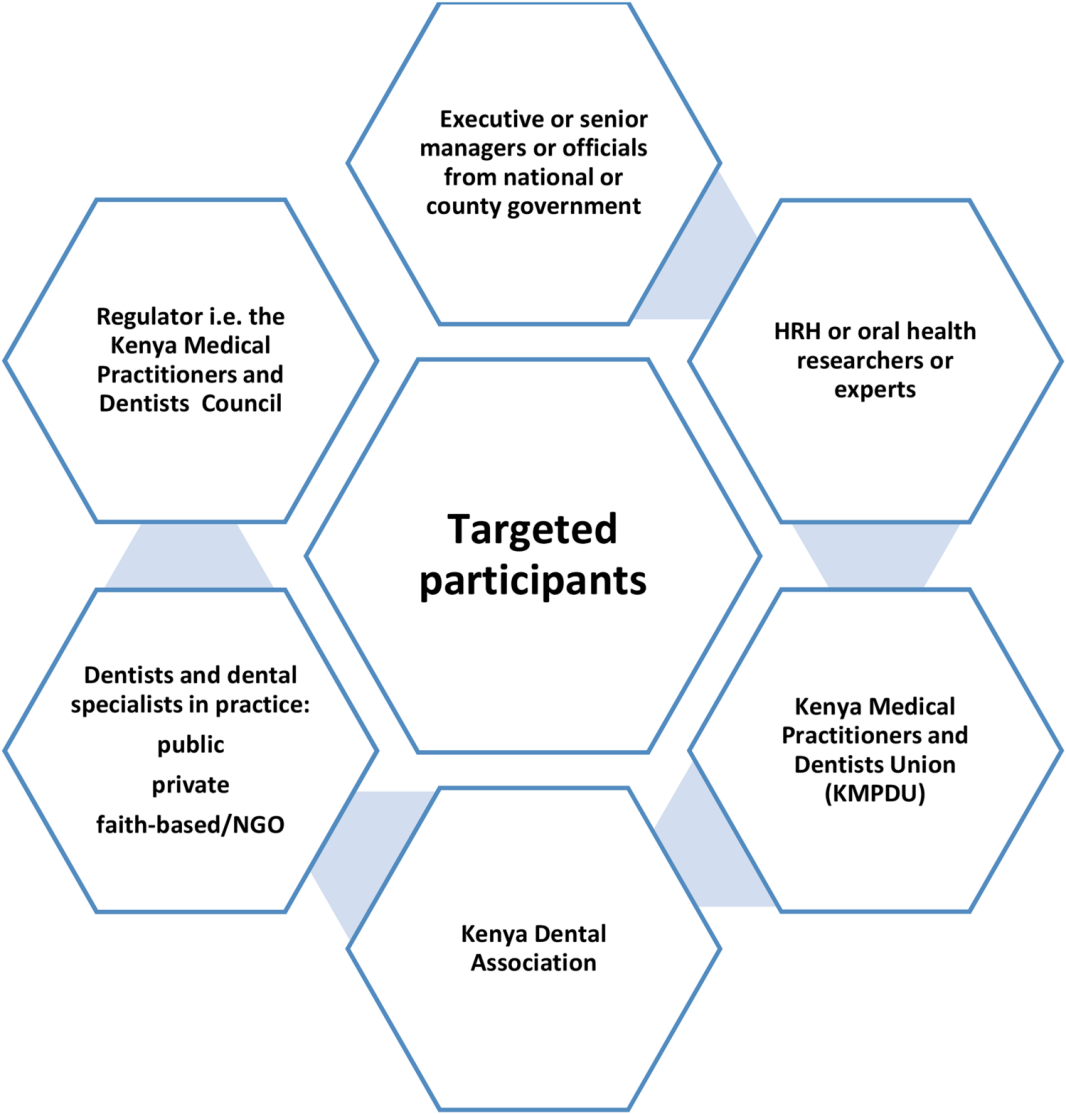
Overview of targeted study participants.

The categories of key informants and dentists selected for interviews are shown in Figure 1.

### Data collection tool

Following an extensive literature review on MJH and in line with the study objectives, the principal investigator (PI = CO) developed two semi-structured interview schedules in English: one for key informants, and one for dentists.

The semi-structured interview schedule for key informants focused on the context of MJH in Kenya among different categories of health professionals; the types or forms of MJH among dentists (generalists and specialists); the factors that influence MJH; the existence of policies or guidelines on MJH; their views on how MJH affects the professional quality of life of dentists and suggestions of specific questions on MJH to include in the planned survey.

The interview schedule with dentists focused on the types or forms of MJH among dentists (generalists and specialists); the reasons for doing MJH; their views on how MJH affects the professional quality of life of dentists and suggestions of specific questions on MJH to include in the planned survey.

The PI piloted the interview schedules with two key informants, and two dentists, to test the clarity of the questions, and the time taken for the interview. In the final interview schedules, two questions were revised to improve clarity. The findings from the pilot study are excluded.

### Data collection

Between November 2022 and January 2023, the PI contacted each participant via email to request voluntary participation in the study. Following informed consent, the PI set the interview date and time with each participant. The PI emailed the information sheet, informed consent forms, and the relevant interview schedule to the study participants.

Due to the COVID-19 pandemic and to minimize contact, the majority of the interviews were conducted online using Zoom or Google Meet, except in three cases where the participants preferred face-to-face meetings. Each interview began with an introduction, assisting the participant to be familiar with the virtual platform and putting the participant at ease. This was followed by an introduction to the study, and an explanation of the voluntary and confidential nature of participation. Prior to the start of the interview, the PI confirmed consent for study participation and for the recording of the interview.

Following informed consent, the PI used the semi-structured interview schedule as a guide to explore each participant's perspectives on MJH among dentists. The PI used probes to obtain details and clarification of responses. Each interview lasted an average of 45 min, but the duration varied depending on the participant's responses.

The PI also made notes after the interviews. All interviews were recorded digitally and labelled with a code. All audio-recordings are kept on a password-protected computer to ensure confidentiality.

### Data management and analysis

The audio recordings were transcribed verbatim. Four transcripts were selected, and the PI (CO) and senior author (LR) analysed the data independently. Each researcher read and reread each transcript independently to ensure familiarization with the data, to get a sense of the whole interview. The transcripts were coded line by line, by writing key words on early impressions, using the direct words from the transcripts. Each researcher made notes on reflections from the data. Following coding stage, each researcher developed themes, interrogated, and evaluated the themes for similarities and differences in meaning across different categories of key informants and the dentists.

Once the process was complete, CO and LR held a meeting to discuss the independent codes and themes, and to reach inter-coder agreement. An iterative process followed of examining the codes and themes in light of the study objectives. Once the codebook was developed by consensus, the PI analysed the remainder of the interviews.

#### Positionality of researchers

CO is a prosthodontist in Nairobi working as a faculty member at a public University dental hospital in Nairobi. Although CO has engaged in MJH previously, he approached the research topic in a non-judgmental manner and adopted a deep listening approach. His experience as a dental specialist encouraged the study participants to elaborate on MJH, their perspectives on its reasons, and the consequences for the health care system.

LR holds a Research Chair on HRH and is the PhD supervisor of CO. LR has no direct association with dentistry in Kenya or with the study participants. The participants received no payment to participate in the research.

## Results

### Characteristics of participants

We interviewed 30 participants: 20 key informants, and 10 dentists. 1 key informant interview was excluded from the final analysis due to the poor audio quality. The 29 interviews included in the final analysis are shown in Table 1.

**Table 1:** Characteristics of study participants.

Qualitative interview/totals	Participants’ sector/background	Male	Female	Total
Key informant interviews	Government health sector policy makers	1	3	4
	KDA	4	0	4
	KMPDU	2	1	3
	Researchers or experts in human resource, health, or dentistry	3	2	5
	KMPDC	3	0	3
In-depth interviews	Dentists (both generalist and specialists)	5	5	10
Total		18	11	29

KDA, Kenya Dental Association; KMPDC, Kenya Medical Practitioners and Dentists Council; KMPDU, Kenya Medical Practitioners, Dentists and Pharmacists Union.

### Themes and sub-themes

The key informant and in-depth interviews with dentists revealed multiple, complex, and overlapping narratives on MJH among dentists. Five themes emerged from the data: (1) MJH as normative practice; (2) Both financial and non-pecuniary reasons drive MJH among dentists; (3) Few or no adverse consequences from absenteeism (4) Dysfunctional and resource-constrained public health sector exacerbate MJH (5) Poor patients and oral health service delivery are collateral damage of MJH.

The themes and subthemes are shown in the Table 2 below. For clarity, each theme is described below.

**Table 2:** Emerging themes and sub-themes from key informant and in-depth interviews.

Theme	Sub-theme
MJH as normative practice	• All professionals engage in MJH • MJH common among dentists • MJH seen as entitlement
Both financial and non-pecuniary reasons drive MJH among dentists	• MJH provides extra income • High rewards from MJH • Ability to practise the full scope of dentistry • Socio-cultural reasons
Few or no adverse consequences from absenteeism	• No or few adverse consequences from absent dentists. • Lack of regulation
Dysfunctional and resource-constrained public health sector exacerbate MJH	• Low budget for oral health care • Lack of prioritization of oral health • Insufficient or lack of equipment and consumables • Poor or no management of MJH
Poor patients and oral health service delivery are collateral damage of MJH	• Long waiting times for poor patients • Compromised or inequitable oral health service delivery

#### Theme 1: MJH as normative practice

Both the key informants and the dentists interviewed indicated that MJH is common among health workers in Kenya, especially among those with specialist qualifications. MJH is reportedly so common that those health workers who do not engage in MJH are considered to be in the minority. One KI stated the following:

“Well, this trend is like everywhere…. not just Nairobi …. and amongst all the professions, especially in the healthcare sector…. even the nurses and medical officers, they're doing the same. What I’ve observed is that clinical officers, nurses, pharmacists, and even the Pharm techs [pharmacy technicians] ……they're also moving about to have multiple engagements” (KI 9, KMPDU official).

In this context, both the key informants and the individual dentists indicated that MJH is common among dentists in both the public and private health sectors in Kenya, among both generalists and specialists, and across the lifespan. Referred to as “locums”, MJH among dentists is seen as the norm, because other health professionals also engaged in it, illustrated by the following quotes.

“In terms of dentists, almost all dentists that I know, and I've interacted with do more than one job. They have a primary job and then they work elsewhere or practise somewhere” (IDI 2, dental specialist).

“It [MJH] is the norm and I can say that because the norm was [to] finish BDS [Bachelor of Dental Surgery] as soon as you can, start to do internship, start locuming” (IDI 6, dentist).

The normative practice of MJH was also highlighted by the union that represents medical doctors and dentists, and by a senior manager in the Kenyan government.

“The term that I hear is [that] dentists are just notorious…… [they] just don't come to work” (KI 1, KMPDU official).

“Most young dentists, when they report to their stations, they find that it's [MJH] the culture, and they continue with it” (KI 3, KMPDU official).

“It [MJH] is a common practice… a very, very common practice. And it has been a common practice for the many years that I’ve practised” (KI 16, Government manager).

Another key informant reported that the norm of MJH is evidenced by the widespread perception that those professionals who do not engage in MJH are seen as “not very intelligent or they lack ambition”. Notwithstanding MJH as normative practice among dentists, the study participants pointed out that dental specialists, especially senior ones, engaged in MJH as an entitlement, and delegated most of their work in public facilities to junior dentists. These senior specialists offer their services on certain times and days of the week, and in some instances, they have admission rights in some hospitals. The latter refers to the explicit permission or special consultancy arrangements to access, admit and treat patients at a particular hospital. One of the key informants highlighted the duality between MJH and dental specialization.

“They [specialists], feel that they needed to specialize in order to have some consultancy arrangements. And these could be at their own practices, or at facilities or fellow dentists’ practices” (KI 12, KMPDC official).

A dental specialist explained MJH as a logical step following his specialization.

“I did partner with my wife, and we opened a clinic in town. I would say, between [year X] to date, I have operated between the three places: the Y doctors plaza, the clinic in town, and H Hospital [primary employer]. The work in H Hospital has changed a bit because now I work primarily as a [specialist]. So, I focus on the theatre dates that are specifically mine and the clinic days seeing patients in the clinic as well. I currently have admission rights in three different hospitals. So, I go as when I have cases that I can attend to in these areas or if I am called to attend to [specialist] cases from these areas. So that in a nutshell, is the [MJH] journey by balancing [my specialty], general dentistry, and work at H [primary employer]” (IDI 2, dental specialist].

#### Theme 2: both financial and non-pecuniary reasons drive MJH

Both the key informants and the in-depth interviews with dentists suggest that financial reasons were the primary driver of MJH, with high financial rewards and a range of non-pecuniary benefits.

A complex and inter-related set of financial reasons drive MJH among dentists. Poor public service remuneration and the high cost of living combined as a powerful driver for MJH as it enables dentists to supplement their government salaries. At the same time, dentists appear to enjoy the stability of a regular monthly income from public service, which they get regardless of the number of patients seen.

The financial benefits from MJH consisted of two main sources, namely investments in dental clinics and compensation for dental professional activities. The study participants highlighted the good returns on investment in dental clinics, especially in Nairobi. Apart from the good return on financial investment, dentists in private practice allegedly received high compensation for their efforts, which makes MJH very attractive.

“The ones who do locum, the reward is quite high. We [dentists] will go and work for an hour and make quite a bit of money” (KI 16, Government manager).

In some instances, dentists use MJH as a stepping stone into full-time private practice. This is because MJH exposes dentists to the running of private practices, building their brand, and a clientele base. Two participants explained as follows:

“You see if you are in one clinic, guys will not know you, but now if like now I am in [X] private hospital, the guys know there is a specialist in [X] private hospital. If I am in [T] public hospital and in [G] town], everyone knows there is this specialist. There is a way this [MJH] builds your name and brand” (IDI 6, Specialist Dentist).

“So, once you're known, after moving around a lot, then once you set up [own private practice] you are confident you will have a few patients here and there” (KI 13, KDA leader).

There are also non-pecuniary reasons for MJH among dentists in Kenya, notably the ability to practise dentistry without limitations, insufficient prioritisation and/or underfunding of oral health services, lack of equipment, and dental materials. These constraints influence the type and range of services dentists offer in the public health sector and contribute to MJH.

“You have a government that will post a dentist, to a hospital where there are no facilities at all for that dentist to practise……. are they finding fulfilment in the career they chose? And the answer is, if you can't find the fulfilment there, then people will try to find it elsewhere” (KI 2, Oral health expert).

“Being able to work in some of these private clinics, at least I'm able to practise what I have learned. The government job may not give me an opportunity to practise everything, to practise what I have learned” (IDI 1, Specialist dentist).

“In the public sector, there are many deficiencies in terms of infrastructure, materials…. If you want to do a root canal, you find you're still using the old system and the old ways of doing treatment. But in private practice, there are newer technologies, there are newer equipment, so you're advancing with the time. So, you're basically growing” (IDI 3, Specialist dentist).

Lack of equipment and materials deprive dentists of an opportunity to practise or improve their skills. MJH provides an opportunity to work in private facilities where they can practise dentistry without limitation and have access to new equipment and sufficient materials.

“I've seen that majority of the dentists, whether they're based in public sector or private, engage in multiple job holding as a means of making additional income. For the public sector, they perhaps feel disenfranchised in their stations, that they're not able to perform the procedures that they were trained for. It [MJH] could be because of a number of reasons, lack of equipment, maybe the patient flows, the patients are not aware of the procedures that they can offer and also the cost. So, they [dentists] tend to get other sources of income and also to gain skills in what we're trained for” (KI 13, KDA).

The individual dentists indicated that younger dentists also benefit from MJH by getting mentorship from senior peers and/or colleagues with more experience.

“So, I think the more places you work, it exposes you to different working environments. It offers you some room for growth, especially if you work in clinics where you have a senior colleague who can mentor you, then you get exposed to different work environments, different kinds of practices” (IDI 7, dentist).

MJH is also driven by various socio-cultural factors. Societal expectations of dentists, taking care of members of the extended family, and the individual desire to maintain a certain lifestyle also play an important role in MJH, as reflected in the following narratives.

“If I were to rely only on my salary from any of those places, it is almost impossible to manage many things. And not only for myself. There's also the extended family, sometimes you know, you have responsibility back to your siblings and parents. And that really also means that you have to expand your income bracket to take care of a lot of those issues. All my brothers and sisters, they went to school, and I in one way or the other, participated in their school fee payment and things of that sort. So, I think there is always a need to expand the income bracket so that you're able to cater for many things that arise not only with for myself, but also just the extended family” (IDI 2, specialist dentist).

“Yeah, if they [dentists] held one job and one salary on one income, they are very unlikely to really meet their needs. Because you can sample what the lifestyle is like. Somebody wants to drive the latest, the high-end model of vehicle, they want to live in a good neighbourhood, they want their kids to go to the costlier schools and all that kind of thing they want to visit social joints that are highly rated. And if you were to just take the average [government] salary, that [lifestyle] may not be attainable. So that I think is primarily what drives most of these multiple job holdings” (KI 4, researcher).

Moreover, there is a common belief that it's a good idea to have a “side hustle” even if employed. The belief that a government salary alone can't make one rich or comfortable is widely held and that accumulating wealth could offer social security, as highlighted by one study participant:

“I think we are in a society which we must always look for more in order to secure our lives and our future. We don't have social security……most people believe that if they can acquire (money) and amass as much as possible, then that would be their security” (KI 2, KMPDC official).

#### Theme 3: few or no adverse consequences

There was consensus among the participants that there are few or no consequences from absenteeism that arises from MJH. In contrast to other health professionals, key informants noted that dentists work primarily in ambulatory care settings and could be absent from work without fatal consequences for patients.

“You can sneak from your workplace to go and do something else and nothing very fatal or critical happens to the client that you were supposed to attend to” (KI 3, Government Manager).

“So, it [MJH] is there. I think the whole of the health profession, not just the dentist. But because of that lack of very critical commitment for a dentist to be at a particular place, I think it [MJH] is more with dentists. It could just be my thought but that is what I feel” [KI 17, Government Policymaker].

Some participants highlighted the lack of regulations on MJH as a constraint that enabled the perceived unfettered practice. While they recognised the importance of remuneration and job satisfaction, they noted that “*very strict regulations, and ways of making sure that people are at their workplace*” would also curtail MJH.

#### Theme 4: dysfunctional and resource-constrained public health sector exacerbate MJH

Health is underfunded in Kenya, with less priority afforded to oral health. The health budget is small, most oral health conditions may not be life threatening and hence those physical health conditions, especially those that are life-threatening, take priority. This is evidenced by the minimal cover for oral health services in the National Hospital Insurance Fund (NHIF). An official from the professional association explained the efforts of a multi-sectoral agency to engage the Ministry of Health to include a broader range of oral health services in the basic insurance package, as most patients are not covered for dental services in the NHIF. In addition, most decision-makers either lack awareness or have a poor attitude towards oral health, and oral health professionals are underrepresented in senior health management. These issues tend to disadvantage oral health in terms of resource allocation.

“I can say the funding has really gone so bad and most public entities are really run down in terms of the infrastructure in there and the staffing and all that” (KI 3, oral health researcher.)

“Dental [oral health] is always placed last, so, even when resources are being placed by the time they come to dental, they are very meagre” (KI 4, KDA official).

The resource constraints in the public health sector exacerbate MJH, while the resultant absenteeism of dentists reinforces the lack of prioritisation or resourcing of oral health services, as highlighted by a senior government manager.

“I think it's only dentists who don't sit at their workstations, even at the facilities at the public institutions. Because you'll see nurses there, you will see the pharmacists and the pharm techs (pharmaceutical technologists) to a very big extent working through the day. Dentists…. very sad thing. And no wonder the county management never sees the need for equipping dental clinics or even expanding the infrastructure. Because the dentists are never seen, they will not present themselves to argue their case” (KI 17, government policy maker).

Moreover, there is poor or no management of MJH by most employers, especially in the public sector, and there is neither a law prohibiting MJH nor any regulations to guide the practice. The KMPDC as the regulator has left the responsibility of supervising dentists to employers, with one of its officials noting that:

“It is the employer who is supposed to know who is supposed to be on duty at what time.” (KI 14, KMPDC official).

Employers in the public sector often lack the skills or the capacity to supervise dentists effectively. Consequently, participants were of the opinion that MJH among dentists is exacerbated by poor supervision in the public sector, illustrated by this key informant comment.

“… The level of supervision is very low. You find somebody is running a practice in Nairobi literally full time, but they're also a dental specialist in another town, in another county. And they go once a week even. So, the level of official supervision is low, that's why they can afford to go” (KI 6, KMPDC leader).

While working hours are clearly stated in the human resource manuals of most employers, compliance is poor. This may be in part due to the pervasive nature of MJH as managers also reportedly engage in MJH.

In some instances, “bureaucratic supervision” that consists of checking whether health professionals were present, was also considered problematic, as there was no attempt to engage with these professionals and to stress the importance of service delivery to the public.

Complex power dynamics underlies MJH and tend to favour dentists, because they are in short supply, they have scarce skills, and the specialists are held in high regard. Some managers may feel dentists should be retained in the public sector, rather than alienated. One KMPDC official narrated an experience when the Council offered to help a manager to improve on a dentist's work attendance in a public health facility. The health manager refused to act, because of the fear of losing the dentist and told the official the following.

“We have fought so hard to get this dentist. If you take him away, then we don't have [a dentist]. We cannot hire another one. So, what we'll do, we'll internally kind of try and find a balance….” (KI 3, KMPDC official).

#### Theme 5: poor patients and oral health service delivery are collateral damage of MJH

This theme captures the comments from key informants that point to the adverse consequences of MJH for poor patients and oral health service delivery in the public sector.

“I think it [MJH] has disadvantaged the poor. So, anybody who can afford some money to spend in private hospitals or private clinics can get good care. But anybody who is struggling, that can [only] afford care in the public facilities is not likely to access the good care. Yeah, they will just access the bare minimum, because there has been that big tilt favouring the private sector” (KI 3, Oral health researcher).

Resource constraints, lack of equipment, and MJH reportedly contribute to the inequities between the public and private health sectors, and the late diagnosis of certain oral conditions of people dependent on the public sector. One specialist explained as follows:

“On one end, you'll find is the patient has stayed for the longest time with the problem and comes when things are dire, as opposed to the side jobs in private situations where people come very early. It’s a thing that I have experienced all through. Even right now we, it's harder to get a stage four disease on a side job or a private centre than a stage four disease in say the [Government hospital]. It's very rare to find a patient coming with an early lesion in the [Government hospital]. Whereas on the other side, mostly you’ll find the early lesions and they’re quite amenable to early interventions” (IDI 2, dental specialist).

In public sector facilities, the study participants indicated that MJH has led to long waiting times for poor patients, both to access treatment, and on the day of the consultation. Key informants reported that dentists arrive late, leave early, and have “local arrangements” that allow them days off from public facilities.

“I remember there's a case we had once at [Hospital X]. A patient came with a fractured mandible. And they came on a Monday, but the fracture had happened like two weeks before. So, we asked them what happened. Why they're coming so late. So, they said they got injured I think on Monday. Then they were told the dentist doesn't come to work until Thursday. So, they stayed with that mandible like that until Thursday then the dentist came and ordered X-rays. And then even after the X-rays were done, you have to wait for a whole week for the dentist to see you” (KI 1, Oral health manager).

“You find internal arrangements and people are coming [to work], in some places twice a week, in some places once a week. These are clinicians. They are doing a locum” (KI 7, Government manager).

“I know in dentistry, we've had issues for quite a long time about the workforce and performance. People [dentists] not coming for the clinics in good time, clinics being closed or something like that” (KI 15, Human resource officer).

Participants reported that MJH has led to compromised or inequitable oral health service delivery. The private sector has thrived at the expense of public sector, and the rich have better access to oral health services that the poor who rely on public service. One participant described the inequity in oral health service as follows:

“It [MJH] avails more services to the high-class people because they can afford it. But the low class, the middle low class, they may not be able to access these services” (KI 7, Government manager).

Participants were of the opinion that MJH among dentists has adverse consequences for the entire health system, especially the quality of oral health service delivery. In the public sector, dentists are in a rush to finish and move to private jobs, while in the private sector, there is motivation to do more procedures and earn more.

“You have to split your time. You know, for example, by probably two o'clock I need to run somewhere else, I have this number of patients waiting for me. So, you kind of feel like you're not like you're not giving 100%. And you feel pressured to try and hurry so that you also go and attend to these other patients on the other side” (IDI 3, specialist dentist).

“The problem is, because of the short period you have [to attend to a patient], and of course you also want to make more money, some of them tend to do what I would say is more or less not quality work. Because they want to do more in a short time and earn more money, you'll find that they're in a hurry, and the trend cuts across wherever they work” (KI 11, Government manager).

## Discussion

MJH is an important labour market phenomenon, which has hitherto been unexplored among dentists working in ambulatory care settings in sub-Saharan Africa. This qualitative study provides unique perspectives of health policy actors in Kenya on MJH among dentists. The study found that MJH is pervasive and has become the norm, because there are high rewards when dentists engage in MJH, and few or no adverse consequences due to the unique nature of their work in ambulatory care settings. Although our study did not measure the extent of MJH among dentists, several studies have reported a high prevalence of MJH among physicians and nurses, regardless of country context or level of income (7, 24, 28, 32). In Nairobi, Kenya, a 2021 study among medical doctors reported a MJH prevalence of 54% (33), albeit with a small, non-random sample. In the USA, a study found a prevalence of 12.2% among dental hygienists (34). However, no studies could be found that measured MJH among dentists, and further studies are needed to quantify the extent and determinants of MJH, especially among dentists in Kenya.

The study participants highlighted the need for additional income as the primary motive for MJH among dentists in Kenya. As is the case for other health professionals, MJH is used by dentists to supplement their income, and to deal with the financial difficulties or the increased financial commitments in their households. The dentists in the study highlighted their financial commitments, as well as the desire for “a good life” after hard work and many years of study, which they cannot afford with their public sector salaries. Other studies have also found that additional income is the primary motivation for MJH (13, 27, 35, 36). In Kenya, compensation in the public health sector is fixed. Poor salaries have been shown to influence the retention of health professionals in the public health sector (37–40). Hence our study findings suggest that there needs to be a health policy discourse on mechanisms to improve salaries for dentists in the public health sector.

Although additional income is the primary motivation for MJH, the resource-constrained and dysfunctional public sector, the lack of continuing professional development, and an inability to practice dentistry without limitations also influence MJH among dentists in Nairobi, as well as their perceived job satisfaction. The under-investment in oral health is illustrated by the less than 1% of the health budget allocation (41, 42). Additionally, oral health services are poorly covered by the national social health insurance package (41, 43). Consequently, the public sector budget is unable to fund modern dental equipment, and consumables, which are essential to the work of dentists. Other studies, albeit among medical specialists have also found that lack of or non-functional equipment contribute to MJH in the private health sector (44, 45). The study participants were of the opinion that poor patients and oral health service delivery are collateral damage of MJH, and that MJH may exacerbate health inequities between poor people dependent on the public health sector, and wealthier individuals who are able to afford private insurance. The development and implementation of an essential equipment and materials list for public dental facilities may be one of the strategies to manage MJH among dentists in Nairobi, Kenya. Furthermore, the availability of such essential equipment and materials in the public sector, is also likely to improve oral health care delivery, especially for poor people.

Many study participants revealed the poor management of health professionals in general, and of MJH in particular. Although other studies have pointed to the poor capacity to manage HRH in many LMICs (46), study participants pointed to the unequal power dynamics between supervisors or manages of health facilities, and dental specialists, which tend to favour dentists. Supervisors are often unwilling to reprimand or discipline dentists, who are never seen because they engage in MJH, even when it affects health care delivery. Unequal power relations have also been found to affect management of absenteeism adversely in LMICs (47). In contrast, supportive supervision could contribute to effective management of MJH (48). Further studies are needed on how different supervisory, or management styles influence MJH among dentists and other health professionals.

The individual dentists interviewed expressed both a desire for status and recognition and societal pressure to maintain a certain lifestyle and indicated that these issues influence MJH. Other studies have also found that cultural attitudes about health professionals, and individual professional identities play a role in MJH (27, 49). Although the role of altruism has been described in improving commitment of health workers to their work (50), others have pointed to the reality of meeting family obligations (51). The study participants highlighted the need to support or meet the expectations of extended family as one of the MJH drivers. Other quantitative studies have found that the number of dependents is associated with MJH (52, 53). Further studies among dentists are needed to determine whether there is a positive association between MJH and the number of dependents.

The vibrant private sector for dentists in Nairobi provides opportunities for multiple sources of income and enables dentists to achieve both status and recognition and to earn lucrative salaries to enhance their lifestyles and meet their financial needs or obligations. At the same time, participants were of the opinion that the private health sector negatively affects the provision of oral health services in the public sector. This happens through the skimping of care that individual dentists provide to poor public sector patients, often rushing to their private practices before they have fulfilled their obligations in terms of hours worked. Study participants also pointed out that MJH influences the attitudes of dentists towards public service, often using it as a stepping stone to start their own private practices. This has also been found in other studies among medical specialists (54, 55).

The interviews revealed that the lack of regulation on MJH compounds the practice. Globally, the regulatory policy options include a total ban on MJH, various restriction of MJH either by hours or income, and explicit regulatory provision under certain conditions as is the case in South Africa (56–58). In resource-constrained settings, bans are either not enforced or they lead to a brain drain and/or a rise in informal payments in the public sector (57). A study in South Africa found that although MJH is allowed provided that prior permission has been obtained, MJH tends to occur without the necessary authority (16). In Kenya, while there is no law against MJH, most employment contracts in the public sector stipulate the minimum hours of work per week. Furthermore, most health workers in the public sector earn a non-practicing allowance (59). This allowance is to caution public sector health workers from forgone income form MJH, the assumption being that they do not engage in it.

The limitations of the study include the urban study setting, the relatively small number of interviews conducted, and potential for social desirability. Although Nairobi is of strategic importance to Kenya, its urban nature means that the results cannot be generalised to the other 46 counties or to rural settings. We interviewed 29 health policy actors, of whom ten were dentists. This is a study limitation as the data collected is representative of their perspectives at a point in time and cannot be generalised to all dentists in Kenya. As social desirability was identified as a potential limitation of the study, the PI ensured all the participants of the confidentiality and anonymity of the information provided.

Our study has numerous strengths. This is one of the first qualitative studies to explore MJH among dentists in an African and an ambulatory care setting. The diverse perspectives of multiple stakeholders add to the body of literature on MJH among health professionals. We obtained rich narratives on MJH and its drivers among dentists, which will be explored and measured in a subsequent quantitative study.

Kenya faces both a shortage of dentists (60) and a maldistribution of dental specialists (61), both which affect the achievement of UHC. Amidst these challenges, MJH could further threaten the achievement of UHC, as has been found in other studies (19, 62). At the same time, MJH could assist with the retention of health workers in the public health sector (63). The high reported occurrence of MJH requires a multi-pronged approach that combines individual, system, and structural interventions. At the individual level, interventions could include open communication with dentists on the ethical dimensions of MJH, the need to prioritise public sector patients, and their suggestions of strategies to create an enabling practice environment for oral health care. At the health system level, there needs to be prioritisation of oral health through appropriate budgeting, short-and medium-term planning in consultation with dentists for the purchase or refurbishment of infrastructure and equipment, and budgetary provisions in line with the plans developed. Structural interventions could include the creation of a forum of all relevant stakeholders (the regulator, professional associations, managers, and dentists) to discuss MJH among health professionals and strategies to ensure that policies meet the twin goals of health system strengthening and retention of scarce skills in the public health sector.

## Conclusion

The diverse perspectives of key health policy actors shed light on the complex phenomenon of MJH among dentists in Nairobi, Kenya. This exploratory, qualitative study found that MJH among dentists in Nairobi, Kenya is common, with high rewards and few or no adverse consequences. Both financial and non-pecuniary reasons drive MJH among the dentists. A dysfunctional and resource-constrained public health sector and lack of regulation exacerbate MJH, while a strong private health sector provides opportunities for multiple sources of income for dentists. The interviews suggest that MJH negatively affects the provision of oral health services in the public sector. We recommend structured engagement among policy actors on MJH among dentists in Nairobi, Kenya to mitigate the potential negative consequences of MJH for patients, the health workforce, and oral healthcare delivery in Nairobi.

The study provided rich, context-specific information on MJH among dentists in an African setting and adds to the growing evidence on this labour market phenomenon among health professionals.

## Data Availability

The raw data supporting the conclusions of this article will be made available by the authors, without undue reservation.
